# Adherence to a reliable PJI diagnostic protocol minimizes unsuspected positive cultures rate

**DOI:** 10.1186/s12891-021-04431-1

**Published:** 2021-08-02

**Authors:** Daniel Pérez-Prieto, Pedro Hinarejos, Albert Alier, Lluïsa Sorlí, Santos Martínez, Lluís Puig, Juan C. Monllau

**Affiliations:** 1 Department of Traumatology and Orthopaedic Surgery, Hospital del Mar – Universitat Autònoma de Barcelona (UAB), Barcelona, Spain; 2 Catalan Institute for Traumatology and Sports Medicine (ICATME), Hospital Universitari Dexeus. – Universitat Autònoma de Barcelona (UAB), Barcelona, Spain; 3 Department of Infectious Diseases, Hospital del Mar – Universitat Autònoma de Barcelona (UAB), Barcelona, Spain

**Keywords:** PJI diagnosis, Culture-negative-PJI, Unsuspected positive cultures

## Abstract

**Background:**

The aim of the present study was to evaluate the incidence of unsuspected PJI when prosthetic revisions are thoroughly evaluated by PJI dedicated orthopedic surgeon before surgery. The hypothesis is that the incidence of unsuspected PJI is reduced by applying this protocol.

**Methods:**

This is a historical cohort study carried out in one university hospital. The prosthetic revision assessment was carried out in January 2019. From that date on, all patients that were programmed for hip or knee revision (either by an orthopedic surgeon specialized or not in septic revisions) were scheduled for a preoperative visit with the same orthopedic surgeon specialized in septic revisions. The diagnostic algorithm applied was based on the Pro-Implant Foundation diagnostic criteria. Prior to the revision assessment, the indication for joint aspiration was done at the surgeons’ discretion (non-specialized in septic revisions) and the preoperative identification of PJI was also done by a hip or knee surgeon (not specialized in septic surgery).

**Results:**

Based on the PIF criteria, there were 15 infections among the revisions in group 1 and 18 PJI in group 2 (*p* > 0.05). The most interesting finding was that there were 7 patients with unsuspected positive cultures in group 1. That represents 11% of all revisions. No patient in group 2 was found with unsuspected positive cultures (*p* < 0.001).

**Conclusion:**

A thorough PJI diagnostic algorithm should be implemented before prosthetic revision to avoid unsuspected positive cultures.

## Background

According to the reports published in the last decades, prosthetic joint infection (PJI) is one of the most dreaded complications among orthopedic surgeons [1–3]. The reason for this fear is multifactorial. The burden of PJI in developing countries is rising and the costs are among the highest in orthopedic procedures [4–7]. Another cause is the poor results published in some studies in which the success rate is lower than 50% [8]. Moreover, undertreated or untreated PJI usually correlates with bad functional outcomes and even depressed and despondent patients [9]. For all of this, some orthopedic surgeons just look the other way and do not look pro-actively to find and diagnose this complication.

Acute PJI often comes with clear signs and symptoms, making the diagnosis of acute PJI uncomplicated [10]. Moreover, most diagnostic tests demonstrate high sensitivity. Conversely, chronic low-grade infections and aseptic prosthetic failures are often quite similar in terms of the clinical presentation. Therefore, a thorough algorithmic approach that combines both the preoperative and intraoperative results is required to reliably diagnose or rule out PJI [1].

The reality is that some patients with PJI (especially low-grade and chronic PJI) are not diagnosed [11, 12]. Therefore, they go untreated while suffering from chronic pain. Indeed, Jacobs et al. reported an approximately 10% rate “unsuspected” PJI among prosthetic exchange procedures [13]. Some of them were not adequately screened for PJI.

Contrariwise, up to a 90% PJI success rate has been reported in the literature with similar functional outcomes to uninfected prosthesis [1, 14–16]. The reported success rate slightly varies depending on the procedure [17–19]. All those studies follow a thorough work-up, diagnostic criteria and treatment for PJI with better outcomes when the patients are treated by specialists in PJI [20–22].

The aim of the present study was to evaluate the incidence of unsuspected PJI when prosthetic revisions are thoroughly evaluated before surgery by PJI dedicated orthopedic surgeon. The hypothesis is that the incidence of unsuspected PJI is reduced by applying this protocol.

## Methods

**Fig. 1:**
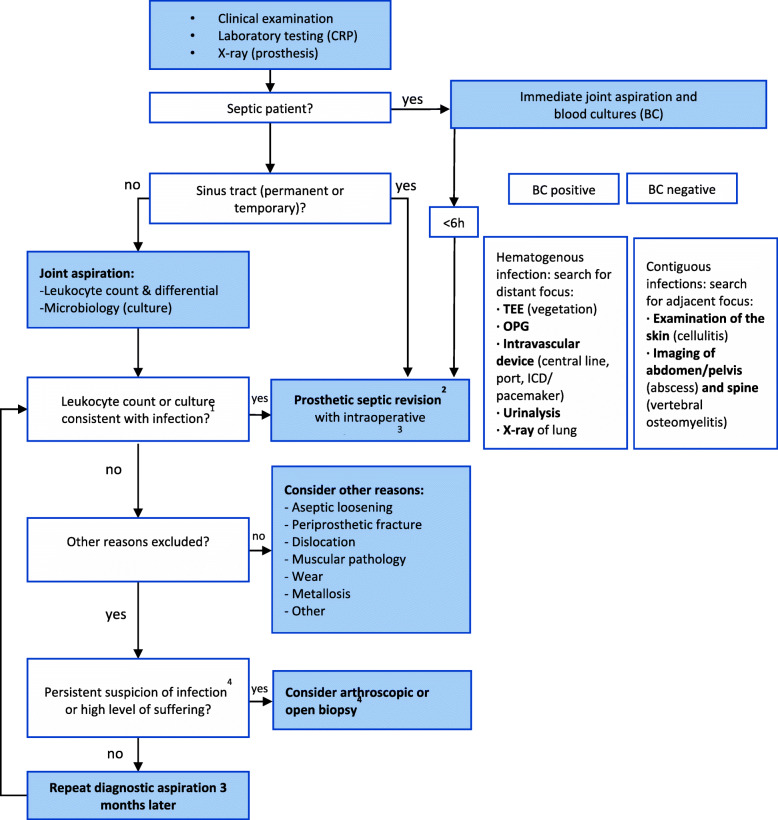
Diagnostic algorithm for PJI. Reproduced with permission from the Pocket Guide to Diagnosis & Treatment of PJI, PROIMPLANT Foundation (version 9, October 2019)

This is a historical cohort study carried out in one university hospital. The study was performed following the Helsinki and Fortaleza protocols. The prosthetic revision assessment was implemented in January 2019. From that date on, all patients that were programmed for hip or knee revision (either by an orthopedic surgeon specialized or not in septic revisions) were scheduled for a preoperative visit with the same orthopedic surgeon specialized in septic surgery (DPP). The group of patients that were thoroughly evaluated before surgery represents Group2. The diagnostic algorithm applied is the one recommended by the Pro-Implant Foundation (PIF) and can be seen in Fig. 1 [1, 23]. This diagnostic protocol was based on the Zimmerli criteria [16, 24]. Recently the European Bone and Joint Infection Society (EBJIS) and the Musculoskeletal Infection Society (MSIS) and the European Society for of Clinical Microbiology and Infectious Diseases (ESCMID) have published the EBJIS definition of PJI, which is based on the aforementioned PIF criteria [25, 26].

A virtual visit was scheduled to revise all medical records. Those records included time of implantation and onset of pain, septic signs at any time (redness, wound healing problems, the presence of sinuses), previous surgeries and the indication for them, loosening on radiographs (time when loosening started and type of loosening), blood test (C-reactive protein -CRP-, erythrocyte sedimentation rate -ESR-) and joint aspiration. When there was any doubt or when the joint aspiration had not yet been performed, the patient was then scheduled for an in person visit.

Prior to this revision assessment, the indication for joint aspiration was done at the surgeons’ discretion and the preoperative identification of PJI was also done by a hip or knee surgeon (not specialized in septic surgery). This group of patients represents Group 1.

The diagnosis of PJI was always done according to the PIF criteria for PJI (which are based on previously published Zimmerli’s criteria) [1, 16, 23, 24, 27]. PJI was considered present when at least one of the following items was fulfilled: (i) the presence of purulence or a sinus tract; (ii) >2000 leucocyte per ml in the synovial fluid or >65% of PMN; (iii) positive hytopathology; (iv) at least 2 positive tissue cultures (or one in case of virulent microorganisms) or a positive sonication culture.

Once the revision assessment protocol was put in place in those cases in which infection had been completely ruled out, a knee or hip surgeon performed the revision. In these cases, 5 periprosthetic tissue cultures, sonication and histopathology samples were collected. Intravenous antibiotic prophylaxis was administered prior to surgery as it does not impact on intraoperative cultures, but no antibiotic treatment was maintained after surgery [27–29]. In doubtful cases (e.g. early loosening, unaccountable pain and punctio sicca, etc.), a similar protocol was followed with some recommendations from the septic surgery team. They involved maintaining postoperative antibiotic therapy until cultures and histopathology were available, carrying out a thorough debridement (like a one-stage exchange) and using antibiotic loaded cement in the case of a cemented prosthesis.

When the diagnosis of PJI was confirmed, surgery was carried out by the septic surgery team and treated accordingly. The debridement performed here was a tumor-like debridement.

### Statistical analysis

Variables are expressed as raw numbers or percentages depending on the case. When 2 related items of data were analyzed, the Student’s t-Test was used. In all cases, a *p* value of less than 0.05 was considered statistically significant. The statistical analysis was done using the SPSS 18.0 (SPSS Inc., Chicago, IL) statistical software package.

## Results

There were 64 prosthesis revisions in group 1 (38 knees and 26 hips) and 69 revised prostheses in group 2 (39 knees and 30 hips).

Based on the PIJ criteria, there were 15 infections among the revisions in group 1 and 18 PJI in group 2 (*p* > 0.05). The details of all infections can be seen in Table 1.

**Table 1:** Table 1 Additional data from patients of both groups

	Type of prosthesis	Microorganism	Protocol fulfillment	Unsuspected positive culture
**Group 1**				
1	THR	CNS	No: sinus tract present. Joint aspiration missing	Yes
2	THR	*Staphylococcus aureus*	No: joint aspiration missing	No
3	TKR	CNS	Yes	No
4	THR	Cutibacterium acnes	No: joint aspiration missing	Yes
5	THR	*Escherichia coli*	No: sinus tract present. Joint aspiration missing	Yes
6	THR	CNS	No: joint aspiration missing	Yes
7	TKR	Escherichia coli	Yes	No
8	TKR	*Enterococcus faecalis* + CNS	Yes	No
9	THR	Cutibacterium acnes	No: joint aspiration missing	Yes
10	TKR	*Pseudomonas aeruginosa*	Yes	No
11	THR	Culture negative	No: joint aspiration missing	No
12	TKR	*Streptococcus viridans*	Yes	No
13	TKR	Cutibacterium acnes	No: joint aspiration missing	Yes
14	TKR	CNS	No: joint aspiration missing	Yes
15	THR	Escherichia coli	Yes	No
**Group 2**				
1	TKA	Pseudomonas aeruginosa	Yes	No
2	TKA	CNS	Yes	No
3	THA	*Candida glabrata*	Yes	No
4	TKA	Staphylococcus aureus	Yes	No
5	THA	Staphylococcus aureus	Yes	No
6	THA	*Enterococcus faecium*	Yes	No
7	THA	*Enterobacter cloacae*	Yes	No
8	TKA	CNS	Yes	No
9	THA	Cutibacterium acnes	Yes	No
10	THA	CNS	Yes	No
11	THA	CNS	Yes	No
12	TKA	Cutibacterium acnes	Yes	No
13	THA	Escherichia coli + CNS	No: aspiration missing	No
14	THA	Pseudomonas aeruginosa	Yes	No
15	TKA	Culture negative	Yes	No
16	THA	Cutibacterium acnes + CNS	Yes	No
17	TKA	*Streptococcus agalactiae*	Yes	No
18	TKA	Culture negative	Yes	No

The most interesting finding was that there were 7 patients with unsuspected positive cultures in group 1. That represents 11% of all revisions. No patient in group 2 was found with unsuspected positive cultures (*p* < 0.001). All these 7 patients required a 2-stage revision within a period of 2–46 weeks.

When it comes to adherence to the diagnostic algorithm, 42 patients in group 1 (65.5%) followed the diagnostic protocol to completion. The most frequent test missing was the preoperative joint puncture (14 patients). There was only one patient in group 2 that did not follow the diagnostic protocol to end (1.4%) (*p* < 0.001). It was due to the surgery being brought forward because of a worsening clinical state.

## Discussion

The most important finding of the present study was that by assessing prosthetic revisions using a strict diagnostic algorithm, the number of unsuspected positive cultures can be reduced. This is an important finding since patients are evaluated prior to surgery and therefore, they are properly treated if the reason for revision is PJI. Otherwise, undiagnosed patients do not receive proper debridement. They may even be discharged without antibiotic treatment.

Any painful prosthetic joint could be a sign of infection and should be thoroughly assessed to rule out PJI. The initial steps to be taken include a clinical examination of the patient and analyses of CRP and ERS as well as plain radiographs. The aforementioned systemic inflammatory parameters are neither sensitive enough or specific to the diagnosis of PJI [11]. Nevertheless, they are easy to acquire and give important information on the systemic repercussion of the infection (in case it exists). They are also important in the PJI follow-up. Therefore, it is important to have them preoperatively to know its evolution.

There are several diagnostic algorithms for PJI. One of the most widely used is the one proposed by the American Association of Orthopedic Surgeons (AAOS) in which they suggest that if a normal CRP and normal ESR are both normal, PJI is discounted [30–32]. However, there are several studies that have proven that CRP and ESR are very non-specific PJI markers and can misdiagnose some 20% of PJI [11]. The most common PJI that those markers do not identify are low-grade infections due to coagulase-negative staphylococci (CNS) and Cutibacterium spp. [13, 23, 33–35]. Moreover, the diagnostic criteria proposed by the Musculoskeletal Infection Society (MSIS) has been found to be less reliable than the European criteria proposed by the PIF in recent studies [23]. For this reason, new diagnostic algorithms have arrived on the scene. One of the most used is the one applied in the present study. It has been employed in several studies for PJI diagnosis in recent years [6, 22, 36]. With the results presented here, it has been shown to be effective at reducing the risk of unsuspected positive cultures in prosthetic revision surgery.

Interestingly enough, the present study has demonstrated that even though both groups were diagnosed applying the same diagnostic criteria, there were 2 patients in Group1 with clear diagnoses of PJI (presence of sinus tract) that were not correctly identified. This is important because an orthopedic surgeon who is not specialized in PJI may misdiagnose infection even though there is a clear sign of it. Moreover, a preoperative joint aspiration was not performed in 9 patients (60%). One reason could be the fact that orthopedic surgeons that are not acquainted with PJI may underestimate the signs of PJI. For instance, in temporary sinus tract, this may occur when the patient is not asked if he or she had a sinus tract at any time.

The risk of unsuspected positive cultures has not been widely studied. A recent study by Jabobs et al. showed that 7.9% of TKA and 12.1% of THA of the supposed aseptic revisions were really PJI [13]. Moojen et al. found that between 4 and 13% of patients with the preoperative diagnosis of aseptic loosening were infected [35]. Similar results have been found in the present study as 11% of “non-controlled” revisions obtained unsuspected positive cultures.

Another important result of the present study is the improvement in PJI management when a dedicated septic surgery team is involved. It is well known how important a multidisciplinary approach to PJI is. Such a team would include orthopedic surgeons, infectious diseases specialists, plastic surgeons and microbiologists [36]. Borens et al. proved that the surgical outcome improved if an orthopedic surgeon specialized in septic surgery performed the PJI revisions. They found a PJI success rate of 83% vs. 61% taking this particular approach to the surgical procedure. Moreover, the economic impact is also relevant. In osteomyelitis cases, Ferguson et al. found that treating infections in dedicated specialist multidisciplinary centers requires a lot of resources and costs are elevated [37]. However, treating infections outside that environment seems to cost more and results in longer patient stays and higher associated costs.

Ideally, PJI should be diagnosed or excluded by performing joint aspiration before revision. Doing so makes for sure planning of the most appropriate treatment strategy. Preoperatively, purulent wound secretion and/or sinus tract communication with the prosthetic joint are confirmative signs of a PJI. In that case, no further diagnostic steps need be taken since it confirms PJI. At this juncture, revision surgery should be scheduled. However, joint aspiration is recommended to try to determine the causative microorganism and its antibiotic susceptibility before revision. This is important to tailoring the antibiotics to the bone cement and/or planning revision in one or two stages. With no confirmatory signs, the most important and sensitive diagnostic measure in the preoperative setting is the leukocyte count of the synovial fluid and the granulocyte differential. Join aspiration must be performed in line with the standard aseptic technique. Culturing of synovial fluid shows low sensitivity as only planktonic bacteria are detected. Most of the bacterial load is embedded in the biofilm on the implant surface and will not be recognized in standard synovial fluid cultures [38]. Synovial fluid leukocyte count analysis is more sensitive. It reflects the host’s reaction to the microorganisms [39]. However, specificity is compromised in situations associated with aseptic inflammatory changes of the joint. Those circumstances include the healing process in the first 4–6 weeks after surgery, inflammatory changes after trauma, recurrent dislocations and in underlying inflammatory arthropathies [39]. Additionally, the optimal cutoff for PJI diagnosis is still subject to controversy. In general, a test with a high sensitivity is preferred at this stage of the diagnostic algorithm. Therefore, a lower leukocyte count (e.g. 2000/μl) is preferred even though it carries the risk of over diagnosis but it avoids missing an infection [1, 25]. The performance of novel biomarkers such as synovial fluid alpha defensin have been assessed in several studies by applying different definition criteria. The results of these studies showed that the leukocyte count is equal to or better than alpha defensin tests and indeed much cheaper [23, 34].

In the case of a dry joint tap, the instillation of saline may lead to a leukocyte count analysis no longer being representative as it dilutes the synovial fluid [39, 40]. If the synovial fluid analysis rules out infection, differential diagnoses should be considered and further investigated (malpositioning of the implant, muscle weakness, tendinopathy, wear, etc.). If the pain remains unexplained, PJI should be still considered. In the case of severe pain with significant compromising of quality-of-life or a loosened prosthesis, revision surgery with meticulous intraoperative diagnostics are advised. A one-stage revision along with a thorough debridement is recommended. Postoperative empiric antibiotic therapy is also recommended until intraoperative microbiology and pathology is available in these cases. In cases when the reason for pain is not clear and the prosthesis is stable and well fixed, biopsy through arthroscopy or mini-open arthrotomy is an alternative. However, the diagnostic yield is rarely significant as the representative area (i.e., the interface between implant and bone) may be difficult to reach. Arthroscopy should be the first option to obtain these biopsies since it is less aggressive. Of course, that depends on the affected joint and the expertise of the treating orthopedic surgeon. Nevertheless, every intervention presents a risk of superinfection. Therefore, they should be avoided as much as possible and reserved for very selected cases, as previously stated. If the pain is satisfactorily managed with analgesics, and the patient does not request immediate intervention, another joint aspiration should be repeated after 1–3 months in order to assess the leukocyte count and granulocyte differential again [1].

The present study has several limitations. The most important is that this method must be proved in a future that is not person-dependent, since the assessment has been made by the same orthopedic surgeon in this study. Another limitation is that the results presented here are related to one specific diagnostic algorithm approach, which prevents us knowing whether other algorithms are equally effective or not.

## Conclusions

The incidence of unsuspected positive cultures may be reduced when a thorough PJI diagnostic algorithm is implemented before prosthetic revision.

## Data Availability

Data and materials are available upon request to the corresponding author.

## Abbreviations

PJI: Prosthetic joint infection
PIF: Pro-Implant Foundation
CRP: C-reactive protein
ESR: Erythrocyte sedimentation rate
PMN: Polymorphonuclear granulocytes
AAOS: American Association of Orthopedic Surgeons
CNS: Coagulase-negative staphylococci
TKA: Total knee arthroplasty
THA: Total hip arthroplasty
